# Factors Affecting the Accuracy of Endoscopic Ultrasonography in the Diagnosis of Early Gastric Cancer Invasion Depth: A Meta-analysis

**DOI:** 10.1155/2019/8241381

**Published:** 2019-12-18

**Authors:** Ding Shi, Xiao-xia Xi

**Affiliations:** ^1^Department of Gastroenterology, Huamei Hospital, University of Chinese Academy of Sciences, Ningbo 315010, China; ^2^Department of Gastroenterology, Henan University of Chinese Medicine, Zhengzhou, China

## Abstract

**Background:**

Endoscopic ultrasonography (EUS) is the first imaging modality for investigating the depth of invasion in early gastric cancer (EGC). However, there is presently no consensus on the accuracy of EUS in diagnosing the invasion depth of EGC.

**Aim:**

This study is aimed at systematically evaluating the accuracy of EUS in diagnosing the invasion depth of EGC and its affecting factors.

**Methods:**

The literatures were identified by searching PubMed, SpringerLink, Cochrane Library, Web of Science, Nature, and Karger knowledge databases. Two researchers extracted the data from the literature and reconstructed these in 2 × 2 tables. The Meta-DiSc software was used to evaluate the overall sensitivity, specificity, positive likelihood ratio, negative likelihood ratio, diagnostic advantage ratio, and 95% confidence interval (CI). The SROC was drawn, and the area under the curve (AUC) was calculated to evaluate the diagnostic value.

**Results:**

A total of 17 articles were selected, which included 4525 cases of lesions. The sensitivity, specificity, positive likelihood ratio, negative likelihood ratio, diagnostic dominance ratio, and 95% CI of EUS for diagnosing EGC was 0.87 (95% CI: 0.86-0.88), 0.67 (95% CI: 0.65-0.70), 2.90 (95% CI: 2.25-3.75), 0.17 (95% CI: 0.13-0.23), and 18.25 (95% CI: 12.61-26.39), respectively. The overall overstaging rate of mucosa/submucosa 1 (M/SM1) and SM by EUS was 13.31% and 32.8%, respectively, while the overall understaging rate of SM was 29.7%. The total misdiagnosis rates for EUS were as follows: 30.4% for lesions ≥ 2 cm and 20.9% for lesions < 2 cm, 27.7% for ulcerative lesions and 21.4% for nonulcerative lesions, and 22% for differentiated lesions and 26.9% for undifferentiated lesions.

**Conclusion:**

EUS has a moderate diagnostic value for the depth of invasion of EGC. The shape, size, and differentiation of lesions might be the main factors that affect the accuracy of EUS in diagnosing EGC.

## 1. Introduction

The incidence of gastric cancer has exhibited worldwide variations. Gastric cancer is a malignant tumor and is associated with a high mortality and morbidity rate. The rank of gastric cancer is 5^th^ in the incidence of malignant tumors and 3^rd^ in the death rate, according to the statistics of the IARC [1]. Therefore, the early diagnosis of gastric cancer is particularly important for the treatment and prognosis of gastric cancer patients.

Early gastric cancer (EGC) refers to gastric cancer that is limited to the mucosal and submucosal layers, regardless of the lymph node metastasis [2]. Gastroscopy has been considered to be the most commonly used for diagnosing EGC. However, this method leads to difficulties in determining the depth of invasion of gastric cancer. Due to the development of endoscopic treatment for EGC, there is a high requirement for investigating the depth of invasion of EGC. Endoscopic submucosal dissection (ESD) and endoscopic mucosal resection (EMR) have become the main standard methods for treating EGC [3, 4]. The lymph node metastasis of EGC is closely correlated with the depth of invasion of tumors [5, 6]. Endoscopic ultrasound (EUS) has been proposed as an accurate method for the locoregional staging of gastric cancer. This method helps in displaying the structure of the gastric wall and has been widely used in the preoperative staging of gastric cancer [7]. At present, various studies have reported the accuracy of EUS in evaluating the invasion depth of EGC. However, these results have revealed a wide level of variability, and the accuracy rate varies between 64.8% and 92% [1, 5, 8–11]. Some studies have concluded that EUS can be used to determine the invasion depth of the mucosal and submucosal layers of EGC, with an accuracy of 80.7%-91.0% [12–18]. In contrast, other scholars have reported that the accuracy of EUS cannot be considered to be more superior to conventional endoscopy and that the accuracy range was within 70-76% for evaluating the mucosal (M) and SM stages of EGC [19, 20]. When EUS was used alone, the overestimation rate of invasion depth reached 18-42% [21, 22]. Several retrospective studies have also reported that EUS has no obvious advantage over conventional endoscopy in predicting the invasion depth, which further raises the question about the clinical significance of EUS for determining EGC treatment strategies [23–25]. The main reason for the differences in these results was that most of these studies have a single-centered design and small sample size. Although few meta-analyses have been published, these did not critically and systemically evaluate the quality of the included literature. Hence, the conclusions were not convincing. Based on the different opinions of researchers and shortcomings of recent meta-analyses on the accuracy of determining the invasion depth of EGC, the present meta-analysis included literatures from 2000 to 2019 and critically evaluated the quality. The present study is aimed at evaluating the accuracy of EUS in diagnosing the depth of invasion of EGC and reassessing the affecting factors, in order to provide a theoretical basis for endoscopic surgery in the treatment of EGC.

## 2. Materials and Methods

### 2.1. Literature Search

A systematic search was conducted in PubMed, SpringerLink, Cochrane Library, Web of Science, Nature, and Karger knowledge databases from 2000 to 2019. The last included article was on April 8, 2019.

The English references of the obtained articles were retrieved. The search terms, which included the subjects and free words, were as follows: “endoscopy ultrasonography”, “miniprobe endoscopy”, “Ultrasonography”, “EUS”, “early gastric cancer”, “early gastric neoplasm”, “early gastric carcinoma”, “early gastric tumors”, “EGC”, “invasion depth”, and “early stage”.

### 2.2. Inclusion Criteria

The inclusion criteria were as follows: (1) articles that are aimed at assessing the accuracy of EUS in evaluating the invasion depth of EGC, with either a retrospective or prospective design; (2) EUS performed in all cases with EGC; (3) postoperative histopathology or biopsy considered as the gold standard for diagnosing EGC; (4) sufficient data available to construct a two-by-two contingency table, in which the cells in the table were labeled as true positive, false positive, true negative, and false negative; and (5) articles published in the English language.

### 2.3. Exclusion Criteria

The exclusion criteria were as follows: (1) reviews, lectures, case reports, and other nonoriginal research literature; (2) EUS performed before preoperative chemotherapy and/or radiotherapy, in order to avoid the confounding effect of the disease; (3) the depth of invasion not confirmed by histopathology in the study; (4) full-text studies that did not include the tables and data unavailability even after contacting the corresponding author by e-mail; and (5) studies that had a total sample size of <30.

### 2.4. Data Extraction

All literature information was independently extracted by two researchers and entered into an Excel spreadsheet. A third party participated when there were any disputes. The following information were extracted: name of the author, year of publication, country, sample size, research type, location of the lesion, shape of the lesion, size of the lesion, differentiation type of the lesion, endoscopy model, ultrasound frequency, gold standard, true positive (TP) values, false positive (FP) values, true negative (TN) values, and false negative (FN) values for the M/SM stages. A 4 × 4 or 3 × 3 and 2 × 2 table of the original data was reconstructed. If the full text of any study was not free, the corresponding author was contacted by e-mail. The basic features are presented in Table 1.

### 2.5. Quality Evaluation

**Figure 1:**
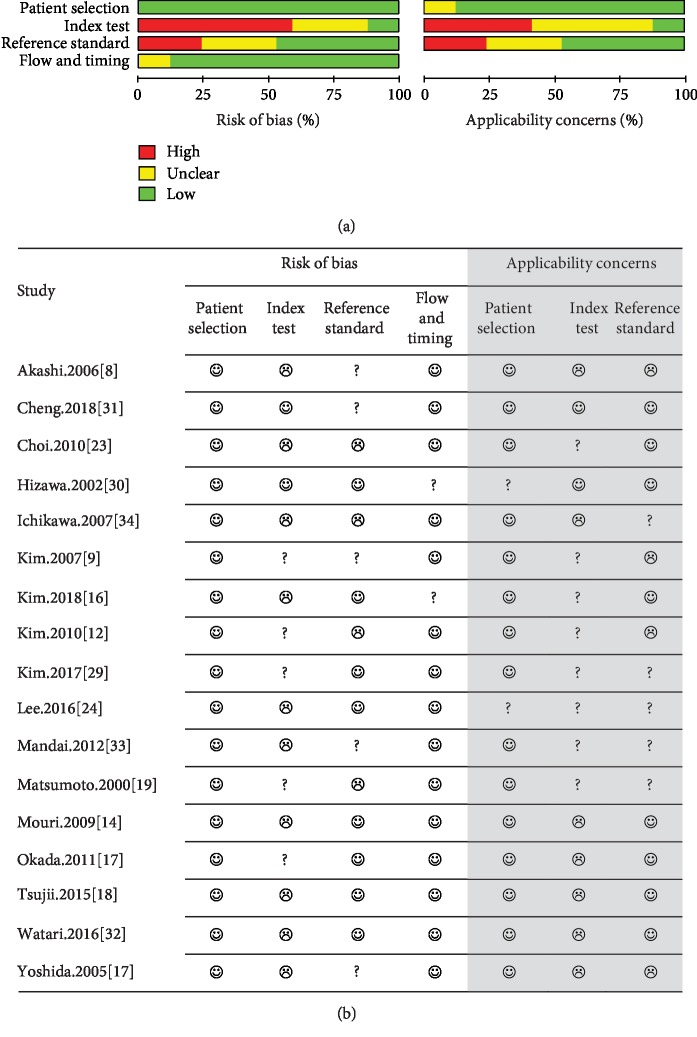
Quality evaluation table. (a) Proportion of studies with low, high, or unclear risk of bias and concerns regarding applicability; (b) methodological evaluation according to QUADAS-2. ☺: low risk; ☹: high risk; ?: unclear risk.

A quality assessment was performed to evaluate the quality of the diagnostic accuracy studies and determine the sources of potential heterogeneity (Figure 1). The quality assessment also identified any risk of bias and applicability concerns. The quality assessment of diagnostic accuracy studies version 2 (QUADAS-2) was used to evaluate the quality of the literature, which basically included four areas: case selection, index test referencing, gold criteria, flow and progress. Each item was assessed for three results: yes, no, and unclear. “Yes” means that the included literature conformed to the content, “no” means that the included literature does not conform to the content, and “unclear” means that sufficient information could not be extracted. The bias in the case selection and clinical applicability was classified as follows: low risk, high risk, and uncertain risk.

### 2.6. Data Analysis

Review Manager 5.3 (The Nordic Cochrane Centre, The Cochrane Collaboration, UK) was used for the quality evaluation. The Spearman correlation coefficient was calculated using Meta-DiSc 14.0 (Unit of Clinical Biostatistics, Ramon y Cajal Hospital, Madrid, Spain) and was used to calculate the sensitivity (Sen), specificity (Spe), positive likelihood ratio (PLR), negative likelihood ratio (NLR), and diagnostic ratio (DOR), with a 95% confidence interval (95% CI). The summary receiver operating characteristic (SROC) curve was also analyzed. The Stata 12.0 software (Stata Corporation, College Station, TX, USA) was used to calculate the prior probability and posterior probability.

The threshold effect was observed when the Spearman correlation coefficient exhibited a strong linear positive correlation. When the Spearman correlation coefficient exhibited a weak linear positive correlation, no threshold effect was revealed. A metaregression analysis was performed to assess the source of heterogeneity. Heterogeneity was evaluated using the *I*^2^ statistic, and *I*^2^ > 50% indicated the existence of heterogeneity. The fixed-effects model was used for the meta-analysis when the *I*^2^ value was <50, while the random-effects model was used when the *I*^2^ value was >50. The meta-analysis test level was *α* = 0.05. Deeks' funnel plot was used to evaluate the publication bias.

## 3. Results

### 3.1. Document Retrieval and Characteristics of the Included Literature

**Figure 2:**
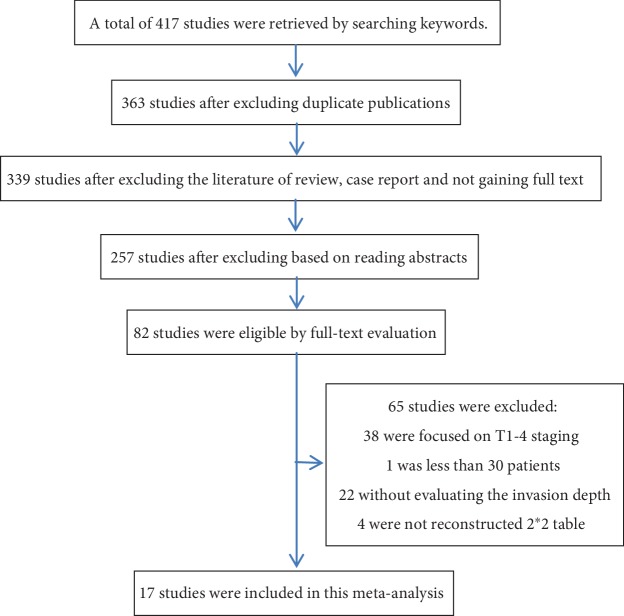
Flow diagram of the studies identified in the meta-analysis.

After the critical evaluation, a total of 17 previously published studies were included from the different datasets. A total of 417 literatures were retrieved by searching keywords from the different databases, and 54 repeated publications were excluded according to the exclusion criteria. A total of 281 articles were excluded, because these articles were reviews and case reports, or the full text was not available. The remaining 82 articles were reviewed and assessed. Among these articles, 38 articles were further excluded, because these did not involve the M and SM invasion depth; one article was excluded, because the sample size of the study was <30 cases; 22 articles were excluded, because the invasion depth was not assessed; and four articles were excluded, because the 2 × 2 tables could not be reconstructed. Hence, after the critical assessment based on the inclusion and exclusion criteria, 17 published studies were included for the present meta-analysis. The process for the literature screening and selection of studies is presented in Figure 2. The included studies were published and conducted by authors from Korea, China, and Japan, in which a total of 4525 lesions were included. In these included studies, the types of research included prospective and retrospective studies. In addition, the frequency range of the ultrasound was 7.5-30.0 MHz.

### 3.2. Quality Evaluation

The QUADAS-2 quality scale was used to assess the bias risk and clinical applicability of the continuous measurements in the included studies (Figure 1). Among the included studies, five studies included patients with preoperatively suspected but postoperatively confirmed gastric cancer [8, 18, 22, 24, 26], while the remaining studies included patients with confirmed gastric cancer. In all the included studies, EUS defined the depth of invasion of EGC as M/SM, while four of these studies defined the threshold of invasion depth as SM1 < 500 *μ* [7, 11, 25, 27]. However, among the 17 included studies, three studies used the blind method, but these studies did not include the endoscopic diagnosis when the sample was sent for histopathology [23, 28, 29]. The remaining 14 studies did not use the blind method but included the endoscopic diagnosis when the sample was sent for histopathology. The nonblinded interpretation of the results may be the reason of high risk in the included literature. The quality evaluation revealed that 17 included articles were of high quality, and none of these had methodological defects.

### 3.3. Statistical Results

**Figure 3:**
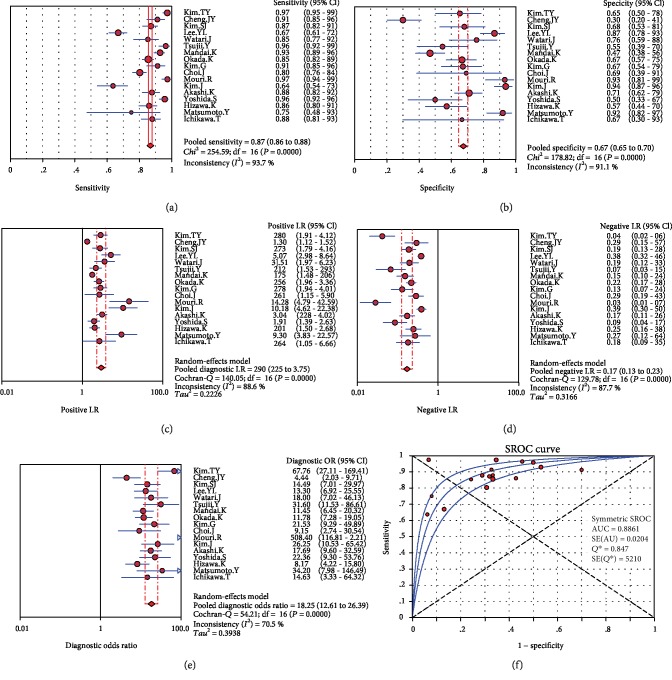
Forest plots: (a) sensitivity; (b) specificity; (c) positive likelihood ratio; (d) negative likelihood ratio; (e) diagnostic odds ratio of EUS for the mucosal/submucosal staging of EGC; (f) SROC curve. CI: confidence interval.

**Figure 4:**
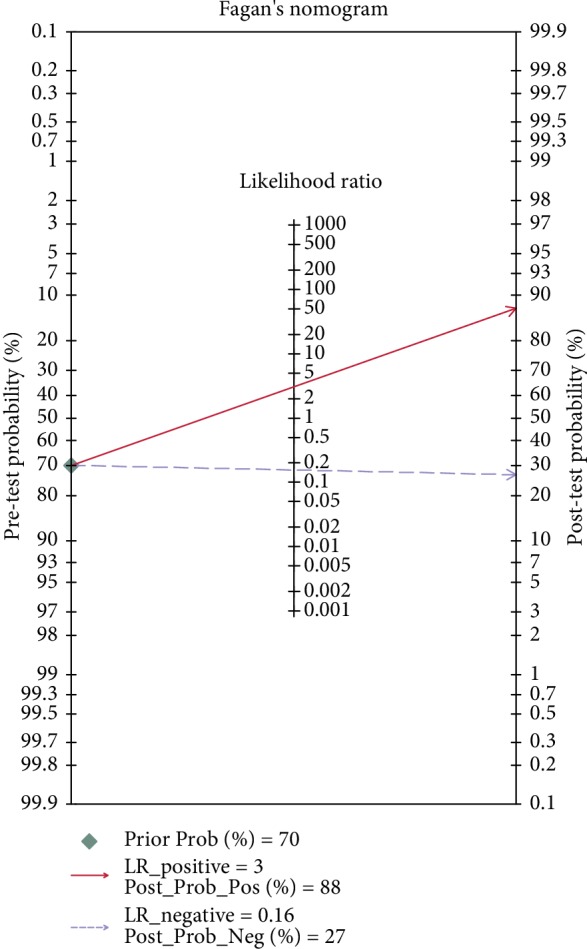
Fagan nomogram in evaluating the overall diagnostic value of EUS for predicting invasion depth of EGC.

In the present study, the random-effects model was used, because the *I*^2^ value was >50%. For the accuracy of EUS in diagnosing the invasion depth of EGC, the aggregate Sen was 0.87 (95% CI: 0.86-0.88, Figure 3(a)), the Spe was 0.67 (95% CI: 0.65-0.70, Figure 3(b)), the PLR was 2.90 (95% CI: 2.25-3.75, Figure 3(c)), the NLR was 0.17 (95% CI: 0.13-0.23, Figure 3(d)), the DOR was 18.25 (95% CI: 12.61-26.39, Figure 3(e)), the area under SROC curve was 0.8861 (Figure 3(f)), and the prior probability and posterior probability were 70% and 88%, respectively (Figure 4).

### 3.4. Metaregression and Subgroup Analysis

The heterogeneity test results (*I*^2^ > 50%) revealed the presence of heterogeneity in the present meta-analysis. However, Spearman's correlation coefficient was 0.540 (*P* = 0.025), which further revealed the absence of any threshold effect. This indicates that the heterogeneous sources of the included articles were not caused by the threshold effect. A metaregression analysis was conducted to determine the heterogeneity beyond the threshold effect. According to the characteristics of the studies, a regression analysis was performed for the conducted research years (2018-2010 to 2009-2000), the research countries (China, Japan, and Korea), and research equipment (miniprobe or radial) (Table 2). The metaregression analysis revealed that *P* = 0.0484 of EUS was <0.05, which may be the source of heterogeneity. Furthermore, the results revealed that there was obvious heterogeneity in the endoscopy models (Table 3). Hence, a subgroup analysis of EUS types was performed. The results revealed that the Sen, Spe, PLR, NLR, and DOR of a miniprobe were 90.00 (95% CI: 88-91), 71.00 (95% CI: 67-74), 2.88 (95% CI: 2.34-3.54), 0.13 (95% CI: 0.09-0.19), and 24.91 (95% CI: 15.36-40.39), respectively, while those for the other types of ultrasound were 81.00 (95% CI: 79-83), 63.00 (95% CI: 59-68), 2.66 (95% CI: 1.65-4.29), 0.27 (95% CI: 0.20-0.37), and 10.77 (95% CI: 6.91-16.79), respectively. These indicate that the Sen, Spe, and diagnostic efficiency of a miniature probe EUS are higher than those of the other types of ultrasonography. The subgroup analysis results for the study types were as follows: the results revealed that the Sen, Spe, PLR, NLR, and DOR of the retrospectively study were 88.00 (95% CI: 86-89), 65.00 (95% CI: 61-69), 2.87 (95% CI: 2.00-4.10), 0.18 (95% CI: 0.12-0.27), and 17.29 (95% CI: 11.14-26.86), respectively, while those for the prospectively study were 81.00 (95% CI: 78-84), 82.00 (95% CI: 76-87), 5.19 (95% CI: 2.22-12.14), 0.12 (95% CI: 0.02-0.60), and 45.88 (95% CI: 8.57-245.55), respectively.

### 3.5. Evaluation of Publication Bias and Result Detection

**Figure 5:**
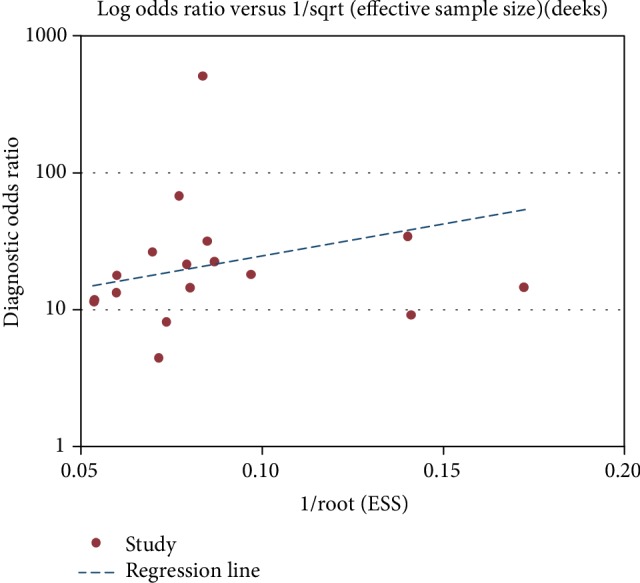
Deeks' funnel plot for publication bias.

Taking the reciprocal of the effective sample size (1/ESS1/2) as the abscissa and the DOR as the ordinate, the funnel map was drawn, and the slope coefficient was calculated. Then, according to the symmetry of the funnel map and slope coefficient, the publication bias was evaluated. The results presented a symmetrical funnel plot, and the slope coefficient was *P* = 0.374, >0.05. This indicates that there is no publication bias between the included studies (Figure 5 and Table 4).

### 3.6. Affecting Factors

The factors that influence the accuracy of EUS in diagnosing EGC invasion depth were evaluated. The main aspects were as follows: (1) Overstaging and understaging: the rate of overstaging of M/SM1 by EUS ranged from 1.3% to 33.1%, and the total overstaging rate in the included literatures was 13.31%; SM ranged from 3.2% to 93.4%, and the total overstaging rate in the included literatures was 32.8%; the understaging rate of SM ranged from 4.3% to 46.0%, and the total understaging rate in the included literatures was 29.7% (Tables 5 and 6). (2) Lesion size: The misdiagnosis rate of EUS in detecting lesions of ≥2 cm in size ranged from 5.2% to 43.8%, and the total misdiagnosis rate was 30.4%. For lesions <2 cm in size, the rate of misdiagnosis ranged from 10.7% to 50.1%, and the total misdiagnosis rate was 20.9%. The misdiagnosis rate of EUS in lesions ≥ 2 cm in size was significantly higher than that for lesions < 2 cm in size, and the significant statistical *P* value was <0.05 (*P* ≤ 0.001, Table 7). (3) Lesion shape: The misdiagnosis rate of EUS in ulcerative lesions ranged from 12.0% to 73.1%, and the total misdiagnosis rate was 27.7%. For nonulcerative lesions, the rate of misdiagnosis ranged from 1.7% to 68.8%, and the total misdiagnosis rate was 21.4%. The misdiagnosis rate of EUS for ulcerative lesions was significantly higher than that for nonulcerative lesions, and there was a statistically significant *P* difference (*P* ≤ 0.001, Table 8). (4) Differentiation type: The misdiagnosis rate of EUS in differentiated lesions ranged from 3% to 36%, and the total misdiagnosis rate was 22%. In undifferentiated lesions, the rate of misdiagnosis ranged from 5% to 46.8%, and the total misdiagnosis rate was 26.9%. The misdiagnosis rate of EUS for undifferentiated lesions was significantly higher than that of differentiated lesions, and there was a statistically significant *P* difference (*P* = 0.0030, Table 9). (5) Lesion location: The misdiagnosis rate of EUS for gastric lesions located on the upper third area ranged from 6.7% to 41.4%, and the total misdiagnosis rate was 24.3%. The misdiagnosis rate of EUS for gastric lesions located on the middle third ranged from 11.6% to 35.6%, and the total misdiagnosis rate was 20.8%. The misdiagnosis rate of EUS for gastric lesions located on the lower third ranged from 14% to 37.4%, and the total misdiagnosis rate was 20.3%. However, there was no statistically significant difference in the misdiagnosis rate for upper, middle, and lower gastric lesions by EUS (*P* = 0.181, Table 10).

## 4. Discussion

Invasion and metastasis remain as great challenges in curing malignant gastric cancers. Therefore, it is significantly important to diagnose gastric cancer at the early stage, in order to initiate early treatment as soon as possible and further improve the 5-year survival rate. Various available treatment modalities of gastric cancer have been dependent on the accuracy of preoperative staging. Endoscopic treatment strategies are based on whether the EGC involves the mucosal or submucosal layers and in detecting the extent of changes of the ultrasonography, such as the determination of depth of invasion. At present, EUS has been considered as the most effective nonsurgical method for assessing primary tumors, with a high diagnostic rate for staging gastric cancer [30]. Studies have concluded that EUS has a high rate of accuracy, when compared to conventional endoscopy. [27]. However, contradictory results have been investigated among various studies, in terms of the rate of accuracy and affecting factors of EUS in the diagnosis of EGC invasion depth. Therefore, there is significant importance to determine the factors that affect the accuracy of EUS in the diagnosis of EGC invasion depth. The results of the present study differed from those in previously published meta-analyses [31], in which the overall Sen, Spe, and diagnostic advantage ratio of EUS in the diagnosis of EGC invasion depth were 0.87 (95% CI: 0.86-0.88), 0.67 (95% CI: 0.65-0.70) and 18.25 (95% CI: 12.61-26.39), respectively. This suggests that EUS has a higher Sen, lower Spe, and better diagnostic effect for EGC invasion depth. In the present meta-analysis, the area under the SROC curve was 0.8861, indicating that EUS has a medium value for diagnosing the invasion depth of EGC. The diagnostic value of EUS was high when the SROC curve presented a value of >0.9, while the diagnostic value of EUS was medium when the SROC curve presented a value between 0.7 and 0.9 [32]. However, the PLR should be greater than 10 to establish the diagnosis, while the NLR should be less than 1 to exclude the diagnosis [33]. The PLR and NLR in the present study were 2.40 (95% CI: 1.63-3.52) and 0.16 (95% CI: 0.10-0.25), respectively, suggesting that EUS cannot accurately diagnose the invasion depth of EGC.

The subgroup analysis results revealed that the Sen, Spe, and diagnostic efficiency of a miniprobe EUS significantly improved and was higher, when compared to other types of ultrasonography, suggesting that the miniprobe is more suitable for determining the EGC invasion depth. A miniprobe is more suitable for small lesions, because it has a high level of frequency and allows the layers of small lesions to be more clearly displayed with high resolution. [34]. Therefore, the selection bias can explain why the miniprobe has higher accuracy [35]. It is a well-known fact that the accuracy of EUS in diagnosing invasion depth is affected by both subjective and objective factors. EUS evaluation is highly operator-dependent [18]. The inappropriate estimation of lesion depth due to the operator's inexperience is one of the subjective factors. The results of the present meta-analysis revealed that EUS not only presented with overstaging rates for M/SM1 and SM staging (13.31% and 32.8%, respectively) but also presented with understaging rates for SM (up to 29.7%). In such situations, an underdiagnosis might lead to additional surgery after ESD, while overstaging might lead to overtreatment [36]. Therefore, determining how to prevent overstaging and understaging remains as an important issue. Some studies have revealed that overdiagnosis during EUS occurred with ulceration or fibrosis. Hence, a pattern analysis might be an effective modality for overcoming the limitations of EUS in differentiating SM cancer invasions [23, 27, 29, 37, 38]. The factors that affected the accuracy of EUS in diagnosing EGC invasion depth were further analyzed. The results demonstrated that the lesion shape, size, location, and differentiation type contributed to the misdiagnosis rate of EUS. The misdiagnosis rate of EUS for ulcerative lesions of ≥2 cm in size was significantly higher than that of nonulcerative lesions of <2 cm in size, and the *P* value was statistically significant. Both factors are endoscopic visual factors that may affect the real-time endosonographic judgment [18, 23], suggesting that reviewing EUS imaging, instead of real-time EUS, might reduce the misjudgment of invasion depth [39]. The previous meta-analysis revealed that the influence of lesion location on the misdiagnosis of EUS was dependent on whether the ultrasound probe and target lesion could be immersed in deaerated water at the same time. This problem could be overcome by adjusting the volumes of air and deaerated water [7, 26]. The present study also revealed that the misdiagnosis rate of undifferentiated cancer was significantly higher than that of differentiated cancer. This might be correlated with the different growth patterns of two types of cancers. The diffuse invasion of undifferentiated tumor cells (individually or in small nests) might be the main cause for the misdiagnosis of EUS [40, 41].

In addition, a subgroup analysis of the types of studies included in the literature was performed. These results revealed that the sensitivity of the retrospective study was similar to that of the prospective study, but the specificity of the prospective study was much higher than that of the retrospective study. Therefore, the accuracy of EUS in diagnosing the invasion depth of EGC needs to be verified through prospective studies with a large sample size.

### 4.1. Limitations

The present study had the following limitations: (1) Merely literatures in the English language were included. This might have omitted data from articles published in other languages. (2) The maximum number of included literatures in the present study was obtained from retrospective studies. (3) High heterogeneity was observed in the present study. Furthermore, merely a subgroup analysis on population, sample size, and publication year was conducted. Various unreported factors could affect the overall estimation.

## 5. Conclusion

EUS provides a reliable method for the diagnosis accuracy of invasion depth in EGC, in which a miniprobe might be a better choice. Attention should be given by the operator on the factors that could affect the accuracy of EUS in diagnosing the EGC invasion depth. The shape, size, and differentiation of lesions might be the main factors that could affect the accuracy of EUS in the diagnosis of EGC.

## Figures and Tables

**Table 1:** Characteristics of the included studies.

ID	Country	Sample size	Study population	EUS type	Frequency (MHz)	ECG staging	Reference test
Kim et al. [16]	Korea	345	Confirmed ECG	Miniprobe	20	M, SM1, SM2	Surgery, ESD, or EMR
Cheng et al. [39]	China	205	Confirmed ECG	Radial	7.5 or 20	M, SM1, SM2	Surgery or ESD
Kim et al. [28]	Korea	273	Confirmed ECG	Miniprobe	20	M, SM	Surgery or ESD
Lee et al. [24]	Korea	393	Confirmed ECG	Radial	20	M, SM	Surgery, ESD, or EMR
Watari et al. [42]	Japan	132	Confirmed ECG	Miniprobe	20	M, SM1, SM2	Surgery, ESD, or EMR
Tsujii et al. [18]	Japan	208	Confirmed ECG	Miniprobe	12, 20	M, SM	Surgery or ESD
Mandai and Yasuda [34]	Japan	406	Confirmed ECG	Miniprobe or radial	20	M/SM1, SM2	Surgery, ESD, or EMR
Okada et al. [7]	Japan	542	Confirmed ECG	Miniprobe	20	M, SM1, SM2	Surgery or ESD
Choi et al. [23]	Korea	388	Confirmed ECG	Miniprobe	12	M, SM	Surgery or ESD
Kim et al. [12]	Korea	176	Suspected ECG	Miniprobe	20	M, SM	Surgery, ESD, or EMR
Mouri et al. [14]	Japan	235	Confirmed ECG	Miniprobe	20	M, SM	Surgery, ESD, or EMR
Kim et al. [9]	Korea	206	Suspected ECG	Miniprobe or radial	5, 7.5, 12, 15, 20	M, SM	Surgery or EMR
Akashi et al. [8]	Japan	267	Suspected EC	Miniprobe	12, 15, 20	M, SM	Surgery or EMR
Yoshida et al. [17]	Japan	295	Confirmed ECG	Miniprobe	7.5, 12, 15, 20	M, SM1, SM2	Surgery
Hizawa et al. [29]	Japan	234	Confirmed ECG	Miniprobe or radial	12, 20	M, SM	Surgery, ESD, or EMR
Matsumoto et al. [19]	Japan	78	Confirmed ECG	Miniprobe	20	M, SM	Surgery, ESD, or EMR
Ichikawa et al. [43]	Japan	142	Confirmed ECG	Miniprobe	12, 20, 30	M, SM1, SM2	Surgery or ESD

EMR: endoscopic mucosal resection; ESD: endoscopic submucosal dissection; EUS: endoscopic ultrasonography; M: mucosa; SM: submucosa.

**Table 2:** Metaregression analysis.

Covariate	Coefficient	*P* value	RDOR	95% CI
Publication date (after 2010 *vs.* before 2010)	-0.096	0.8293	0.91	(0.36; 2.32)
Country (Japan *vs.* others)	0.151	0.2343	1.16	(0.70; 1.92)
EUS type (miniprobe *vs.* others)	-0.863	0.0484	0.42	(0.18; 0.99)

CI: confidence interval; EUS: endoscopic ultrasonography; RDOR: relative diagnostic odds ratio.

**Table 3:** Subgroup analysis.

Subgroup	*n*	Sensitivity (%)	Specificity (%)	PLR	NLR	DOR
Overall	17	87 (86-88)	67 (65-70)	2.90 (2.25-3.75)	0.17 (0.13-0.23)	18.25 (12.61-26.39)
Only miniprobe	11	90 (88-91)	71 (67-74)	2.88 (2.34-3.54)	0.13 (0.09-0.19)	24.91 (15.36-40.39)
Radial EUS included	6	81 (79-83)	63 (59-68)	2.66 (1.65-4.29)	0.27 (0.20-0.37)	10.77 (6.91-16.79)
Retrospective study	10	88 (86-89)	65 (61-69)	2.87 (2.00-4.10)	0.18 (0.12-0.27)	17.67 (10.82-28.85)
Prospective study	3	81 (78-84)	82 (76-87)	5.19 (2.22-12.14)	0.12 (0.02-0.60)	45.88 (8.57-245.55)

EUS: endoscopic ultrasonography; PLR: position likelihood ratio; NLR: negative likelihood ratio; DOR: diagnostic odds ratio.

**Table 4:** Slope coefficient.

yb	Coef.	Std. Err.	*t*	*P* > ∣*t*∣	95% Conf. interval	
Bias	10.4976	11.46026	0.92	0.374	-13.92936	34.92456
Intercept	2.147477	8663308	2.48	0.026	0.3009368	3.994018

**Table 5:** The rates of overestimation of M/SM1 by EUS.

ID	Overstaging rate
Mandai and Yasuda [34]	67/327 (20.5%)
Kim et al. [12]	20/125 (16%)
Akashi et al. [8]	12/121 (9.9%)
Hizawa et al. [29]	27/174 (18.4%)
Kim et al. [9]	6/76 (7.9%)
Lee et al. [24]	100/302 (33.1%)
Yoshida et al. [17]	16/243 (6.6%)
Choi et al. [23]	4/305 (1.3%)
Tsujii et al. [18]	20/178 (11.2%)
Mouri et al. [14]	19/166 (11.4%)
Okada et al. [7]	26/370 (7.0%)
Total	317/2387 (13.31%)

**Table 6:** The rates of underestimation and overestimation of SM by EUS.

ID	Overstaging rate	Understaging rate
Mandai and Yasuda [34]	16/79 (20.3%)	20/79 (25.3%)
Kim et al. [12]	10/44 (22.7%)	—
Akashi et al. [8]	11/73 (15.1%)	32/73 (43.8%)
Hizawa et al. [29]	5/60 (8.3%)	26/60 (43.3%)
Kim et al. [9]	—	3/70 (4.3%)
Lee et al. [24]	4/71 (5.6%)	12/71 (16.9%)
Yoshida et al. [17]	1/31 (3.2%)	11/31 (35.5%)
Choi et al. [23]	71/76 (93.4%)	—
Tsujii et al. [18]	6/35 (17.1%)	5/35 (14.3%)
Mouri et al. [14]	—	5/34 (14.7%)
Okada et al. [7]	30/126 (0.8%)	58/126 (46.0%)
Total	154/369 (32.8%)	172/579 (29.7%)

**Table 7:** The influence of lesion size on the depth of invasion diagnosed by EUS.

ID	Diagnostic accuracy of different size lesions				
	<2 cm		≥2 cm		*P* value
	Correct	Incorrect	Correct	Incorrect	
Mandai and Yasuda [34]	217/253 (85.7%)	14.3%	86/153 (56.2%)	43.8%	<0.001
Kim et al. [12]	73/88 (82.9%)	17.1%	69/88 (74.8%)	25.2%	=0.334
Hizawa et al. [29]	125/140 (89.3%)	10.7%	72/84 (77%)	23%	=0.833
Kim et al. [9]	64/93 (68.8%)	31.2%	84/113 (74.3%)	25.7%	=0.043
Kim et al. [28]	183/206 (88.8%)	11.2%	46/67 (68.7%)	31.3%	=0.015
Kim et al. [16]	86/172 (49.9%)	50.1%	17/77 (23.3%)	76.7%	=0.885
Cheng et al. [39]	52/85 (61.2%)	38.8%	86/120 (71.7%)	28.3%	=0.077
Choi et al. [23]	197/223 (88.3%)	11.7%	109/115 (94.8%)	5.2%	<0.001
Total	997/1260 (79.1%)	20.9%	569/817 (69.6%)	30.4%	≤0.001

**Table 8:** The influence of lesion shape on the depth of invasion diagnosed by EUS.

ID	Diagnostic accuracy of different lesion shapes				
	Ulcer		No ulcer		*P* value
	Correct	Incorrect	Correct	Incorrect	
Mandai and Yasuda [34]	59/83 (28.9%)	71.1%	148/171 (86.5%)	13.5%	<0.0010
Kim et al. [12]	51/73 (69.9%)	30.1%	91/103 (88.3%)	11.7%	=0.0030
Akashi et al. [8]	10/20 (50%)	50%	145/165 (87.9%)	12.1%	≤0.0001
Kim et al. [9]	19/29 (65.5%)	34.5%	129/177 (72.9%)	27.1%	=0.5040
Kim et al. [28]	59/74 (79.7%)	20.3%	170/199 (85.4%)	14.6%	=0.0270
Kim et al. [16]	201/228 (88%)	12%	115/117 (98.3%)	1.7%	=0.3040
Yoshida et al. [17]	61/78 (78.2%)	21.3%	204/217 (94%)	6%	<0.0010
Cheng et al. [31]	18/67 (26.9%)	73.1%	43/138 (31.2%)	68.8%	=0.0900
Choi et al. [23]	19/30 (63.3%)	36.7%	287/358 (80.2%)	19.8%	=0.0300
Okada et al. [7]	83/116 (71.2%)	28.8%	426/426 (84.7%)	15.3%	=0.0017
Watari et al. [32]	27/41 (46.7%)	53.5%	104/138 (75.6%)	24.4%	<0.0001
Total	607/839 (72.3%)	27.7%	1737/2209 (78.6%)	21.4%	≤0.0001

**Table 9:** The influence of differentiation type on the depth of invasion diagnosed by EUS.

ID	Diagnostic accuracy of different differentiated types				
	Differentiated		Undifferentiated		*P* value
	Correct	Incorrect	Correct	Incorrect	
Mandai and Yasuda [33]	254/314 (80.8%)	19.2%	49/92 (53.2%)	46.8%	<0.001
Kim et al. [12]	124/145 (85.5%)	14.5%	52/75 (69.2%)	30.8%	=0.020
Hizawa et al. [30]	179/299 (78%)	22%	55/72 (76%)	24%	=0.010
Kim et al. [9]	102/128 (79.4%)	20.6%	104/161 (64.4%)	35.6%	=0.020
Kim et al. [16]	272/425 (64%)	36%	73/114 (64.3%)	35.7%	=1.000
Yoshida et al. [17]	230/256 (90%)	10%	35/40 (87.5%)	12.5%	=0.587
Choi et al. [23]	323/407 (79.3%)	20.7%	65/85 (76.9%)	23.1%	=0.674
Mouri et al. [14]	172/177 (97%)	3%	51/53 (95%)	5%	=0.663
Okada et al. [7]	339/417 (81.3%)	18.7%	105/125 (84.0%)	16%	=0.596
Watari et al. [32]	118/141 (83.9%)	16.1%	35/37 (94.3%)	5.7%	=0.790
Total	2113/2709 (78.0%)	22%	624/854 (73.1%)	26.9%	=0.003

**Table 10:** The influence of lesion location on the depth of invasion diagnosed by EUS.

ID	Diagnostic accuracy of different lesion locations						
	Upper third		Middle third		Lower third		*P* value
	Correct	Incorrect	Correct	Incorrect	Correct	Incorrect	
Mandai and Yasuda [33]	40/52 (76.9%)	23.1%	32/45 (71.1%)	28.9%	231/309 (74.7%)	25.3%	=0.802
Kim et al. [12]	6/8 (75%)	25%	54/68 (79.4%)	20.6%	82/100 (82%)	18%	=0.803
Hizawa et al. [30]	20/32 (63%)	37%	80/105 (76%)	24%	79/92 (86%)	14%	=0.019
Kim et al. [9]	17/24 (70.8%)	29.2%	58/90 (64.4%)	35.6%	73/92 (77%)	23%	=0.082
Kim et al. [29]	32/39 (82.1%)	17.9%	65/75 (86.7%)	13.3%	132/159 (83.0%)	17%	=0.344
Kim et al. [16]	24/34 (70.6%)	29.4%	135/159 (85%)	15%	129/152 (84.7%)	15.3%	=0.034
Cheng et al. [31]	22/30 (73.3%)	26.7%	29/46 (63.0%)	37%	87/129 (62.6%)	37.4%	=0.650
Choi et al. [23]	17/29 (58.6%)	41.4%	43/57 (75.4%)	24.6%	246/302 (81.5%)	18.5%	=0.013
Okada et al. [7]	107/139 (77%)	23%	115/134 (85.8%)	14.2%	222/269 (82.5%)	17.5%	=0.160
Watari et al. [32]	42/45 (93.3%)	6.7%	61/69 (88.4%)	11.6%	29/39 (74.4%)	25.6%	=0.030
Total	327/432 (75.7%)	24.3%	672/848 (79.2%)	20.8%	1310/1643 (79.7%)	20.3%	=0.181

## Additional Points

*Statement of Significance*. EUS provides physicians with very important information on the stage of gastric cancer and has a moderate level of accuracy in diagnosing the invasion depth.
